# Can eukaryotic cells monitor the presence of unreplicated DNA?

**DOI:** 10.1186/1747-1028-2-19

**Published:** 2007-07-10

**Authors:** Jordi Torres-Rosell, Giacomo De Piccoli, Luis Aragón

**Affiliations:** 1 Dept. Ciències Mèdiques Bàsiques, IRBLLEIDA, Universitat de Lleida, Montserrat Roig 2, 25008 Lleida, Spain; 2 Cell Cycle Group, MRC Clinical Sciences Centre, Imperial College London, Du Cane Road, London W12 0NN, UK

## Abstract

Completion of DNA replication before mitosis is essential for genome stability and cell viability. Cellular controls called checkpoints act as surveillance mechanisms capable of detecting errors and blocking cell cycle progression to allow time for those errors to be corrected. An important question in the cell cycle field is whether eukaryotic cells possess mechanisms that monitor ongoing DNA replication and make sure that all chromosomes are fully replicated before entering mitosis, that is whether a replication-completion checkpoint exists. From recent studies with *smc5*–*smc6 *mutants it appears that yeast cells can enter anaphase without noticing that replication in the ribosomal DNA array was unfinished. *smc5–smc6 *mutants are proficient in all known cellular checkpoints, namely the S phase checkpoint, DNA-damage checkpoint, and spindle checkpoint, thus suggesting that none of these checkpoints can monitor the presence of unreplicated segments or the unhindered progression of forks in rDNA. Therefore, these results strongly suggest that normal yeast cells do not contain a DNA replication-completion checkpoint.

## Cell cycle and checkpoints

The cell cycle is the sequence of events by which cells make a copy of themselves, giving rise to two daughter cells at division. The most important events in the cell cycle are the faithful replication and segregation of all chromosomes. In eukaryotic cells these events are separated in time, with every round of DNA replication in S phase preceding chromosome segregation in M phase. Coordination of the different cell cycle phases is dependent upon sequential waves of cyclin/Cdk activity. The cell cycle is thus often viewed as a precise ticking mechanism that drives mitotic cells from one phase into the next one. However, cells have to adapt to different environmental circumstances, as well as to the stochastic nature of most intracellular events. Consequently not all cells take the same time to progress from one phase to the other. In order to adapt to changes in the timing needed to finish cell cycle events, all eukaryotic organisms have special mechanisms, known as cell cycle checkpoints. These prevent cells from leaving one stage until a particular event has taken place, or a particular condition has been satisfied. Failure to delay the cell cycle under some certain circumstances can have disastrous consequences, from genomic instability to the death of the organism.

## Genome replication

Replication origins are licensed during G1 by formation of pre-replicative complexes: the MCM complex, the putative replicative helicase, is loaded in the absence of cyclin-Cdk activity in a Cdc6 and Cdt1 dependent process [1]. During the G1/S transition, activation of S phase cyclins/Cdk and Cdc7-Dbf4 kinases promotes firing of replication origins and the formation of replication bubbles at origins. Two replisomes, headed by traveling MCM complexes, open the DNA double helix as they move in opposite directions. The RFA protein transiently coats the ssDNA, until it is displaced by the advancing RFC-PCNA-DNA polymerase complex [2]. Genome-wide analysis of replication origin activity has shown that origins fire throughout S phase [3,4], and whether an origin will be active or passively replicated in any given S phase depends on its intrinsic efficiency and nuclear context [5]. Replication termination occurs when replication forks traveling in opposite directions meet and the replicated DNA is linked together. Unlike prokaryotes, eukaryotic termination occurs randomly in the intervening regions between replication origins [6]. However, there are several loci where termination is site specific due to the presence of Replication Fork Barriers (RFB), most notably in the ribosomal DNA (rDNA) repeats in *S. cerevisiae *[7,8]. The rDNA locus is located in the middle of chromosome XII and it contains ~150 copies of a 9.1 kb unit. Each copy contains transcription units for the large (35S) and small (5S) rRNA precursors and two intergenic regions (NTS1 and NTS2). Despite the fact that all rDNA copies have an origin of replication only 15% are active at any given S phase [9]. Near the 3' end of the 35S rRNA gene there is a polar RFB. Upon origin activation, bidirectional fork movement proceeds until the leftward-moving fork reaches the RFB site where it becomes arrested [7,8]. Since rightward-moving forks are not affected by the RFB, they replicate through 6–10 rDNA copies until they converge with the leftward-moving fork arrested at the RFB. Therefore, the RFB is a replication termination site. The *FOB1 *gene product is required for RFB activity and it is also necessary for expansion and contraction of rDNA repeat copy number and the production of extrachromosomal rDNA circles (ERCs) [10]. Ribosomal DNA is littered with other factors that impede the progression of replication forks, such as a high level of transcribing RNA polymerases (RNA Pol I). In wild type cells, these factors might be responsible for a small delay in the replication of rDNA compared to the rest of the genome in wild type cells [11,12].

## Replication Fork arrest and the S phase checkpoint

Replication forks can be arrested when nucleotide pools are depleted by hydroxyurea, or when they encounter obstacles such as MMS-induced alkylated bases. Segregation of unrepaired or incompletely replicated DNA can lead to chromosome breakage during mitotic division and genomic instability. To avoid these problems, the S phase checkpoint is activated when the elongation phase of DNA replication is affected. One of the many outcomes of S phase checkpoint activation is a cell cycle arrest that blocks anaphase onset. This mechanism allows cells to adjust the length of S phase in response to replication stress.

A checkpoint gene can be defined as one whose mutation renders cells incompetent for cell cycle arrest under conditions where wild type cells do arrest. For example, the spindle checkpoint delays anaphase until all replicated chromosomes are attached to microtubules from opposite poles and sister chromatids are correctly bioriented. And mutations in any spindle checkpoint gene leads to failures in mitotic arrest upon microtubule depolymerization or in the presence of unattached kinetochores. Analogously to spindle checkpoint mutants, S phase checkpoint mutants are unable to activate the checkpoint when they are treated with HU or MMS, and enter M phase despite incomplete genome replication. As any other signaling pathway, the S phase checkpoint comprises several genes, which can be, not unambigously, grouped into sensors, transducers and effectors [13]. Sensors detect the presence of arrested replication forks; transducers transmit the signal; and the effectors activate the responses, which in the case of the S phase checkpoint range from cell cycle arrest, transcriptional induction of genes needed for repair, stabilization of arrested replication forks and downregulation of late origin firing [14]. It is now well accepted that the main function of the S phase checkpoint is to maintain fork integrity [15].

The main transducers in the S phase checkpoint are the Rad53 and Mec1 protein kinases. However, activation of the S phase checkpoint is started by proteins localized at replication forks. These include proteins required during a normal S phase, such as DNA polymerases (Pol2, Dpb11, Drc1), RFC factors, the Sgs1 helicase and the Top3 topoisomerase [16]. The budding yeast CLASPIN homologue, Mrc1, together with Tof1 and Csm3, also contribute to the initial steps of S phase checkpoint activation. These three proteins are loaded onto replication forks shortly after origin firing and travel with the replication fork during an unperturbed S phase [17]. What is the nature of the signal that engages the S phase checkpoint? Current models, based on HU- and MMS-induced S phase arrest, assume that an increase in the length of RFA-coated ssDNA on stalled replication forks would recruit Mec1-Ddc2 [14]. Mec1, together with Mrc1 and other replication stress sensors already present at the replication fork, would initiate an S phase checkpoint response. Mec1 phosphorylates Mrc1, and both of them are then required to promote Rad53 phosphorylation and activation. Rad53 is then believed to phosphorylate downstream effectors in the pathway. It has been proposed that Mrc1/Tof1 would also prevent uncoupling of replicative helicases from DNA polymerase activity upon replication fork arrest [17], thereby minimizing the length of exposed ssDNA.

During DNA replication, forks can also be slowed down or arrested by DNA secondary structures, specific protein-DNA complexes or collisions between the replication and transcription machineries [18]. These types of replication fork blocks have been described in many loci, such as the rDNA, tRNAs, centromeres and telomeric sequences. Importantly, the S phase checkpoint is not activated when forks are arrested by RFBs in the rDNA, and the checkpoint is dispensable for the stability of these replication fork blocks [19,20]. These observations indicate that replication forks arrested at naturally occurring pause sites do not generate a checkpoint signal, probably due to the absence of significant stretches of ssDNA behind the fork. This implies that forks blocked at natural pausing sites, or ongoing forks that do not generate enough ssDNA, will not launch an S phase checkpoint signal. If these forks are still present at the time of anaphase, they will not prevent chromosome segregation.

## Does the S phase checkpoint account for the flexibility in the timing of DNA replication?

**Figure 1:**
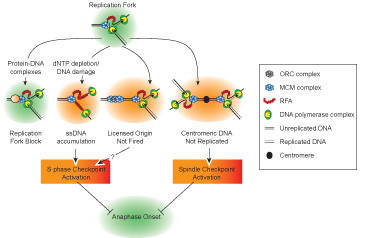
Overview of replication fork conditions that could trigger a checkpoint response. Inhibition of DNA replication by nucleotide depletion or DNA damage, and unfired licensed origins trigger S phase checkpoint activation, whereas unreplicated centromeres could trigger the activation of the spindle checkpoint due to lack of tension at kinetochores. However unreplicated segments of DNA in non-centromeric sites and the presence of forks at natural protein-DNA complexes, like the RFB sites on rDNA genes, do not trigger a checkpoint response.

It is not clear whether ongoing DNA replication during an unperturbed S phase triggers the S phase checkpoint, it is detected by a different mechanism or it is not detected at all. Several hypotheses have been drawn in order to accommodate the alleged flexibility in S phase timing. For example, it has been proposed that a licensed but unfired origin, indicative of incomplete replication, could send a signal to block mitosis [21]. Such scenario would require the presence of ongoing forks, since Dbf4-depleted cells show a reductional anaphase despite the fact that they assemble pre-RCs on origins [22,23] (Figure 1). Another possibility is that unreplicated centromeres would activate the spindle checkpoint, as they cannot be bioriented (Figure 1). Although this mechanism of ongoing replication control might operate occasionally, it would be inefficient because of its blindness to replication forks away from centromeres (Figure 1). Another possibility is that a certain threshold in the number of ongoing replication forks is required to induce the checkpoint. Experiments using Origin Recognition Complex (ORC) mutant *orc2-1 *have demonstrated the presence of a threshold for checkpoint activation during S-phase in response to MMS damage [24]. But still they do not preclude the possibility that a catastrophic anaphase can be triggered in the presence of a few undamaged replication forks (Figure 1). Other authors have proposed that ongoing replication forks would activate the DNA damage checkpoint protein Rad9 [25]. This is based upon the finding that a small but significant population of cells from a strain bearing a yeast artificial chromosome (YAC), with five out of eight origins deleted (5ORIΔ), entered a second cell cycle with kinetics somewhat slower than controls, indicative of checkpoint activation. Importantly, it was not shown if the 5ORIΔ strain would also delay anaphase onset. The authors suggested that Rad9, a sensor of the DNA damage response not involved in S phase checkpoint activation, would be required to lengthen S phase to allow replication completion. This is based on the observation that the 5ORIΔ chromosome is lost 10-times more frequently in a *rad9 *mutant background [25]. However, the strain bearing the 5ORIΔ YAC does not phosphorylate Rad53, indicating that there is no checkpoint activation [25]. And there is no report indicating that, in the *rad9 *mutant, the 5ORIΔ YAC does no longer delay the cell cycle. It is tempting to speculate that 5ORIΔ cells could also delay entry into the next cell cycle as a result of anaphase entry and chromosome breakage in the presence of replication forks. Other reports point towards a lack of replication completion checkpoint. *sic1Δ *mutants cannot inhibit cyclin-Cdk activity and display a shorter G1 phase. The shorter G1 seems to prevent the establishment of a consistent number of pre-replicative complexes and *sic1Δ *mutants start replication from fewer origins [26]. As a consequence *sic1Δ *mutants enter anaphase with incomplete genome replication. Importantly, they do so with no checkpoint activation [26], suggesting that ongoing replication is not detected. In striking contrast, failure to activate late origin firing in *clb5Δ *mutants seems to have the opposite effect, and *clb5Δ *cells display anomalous Rad53 activation, even in a G1 arrest [27].

In any of the cases reviewed above, there is no direct experimental data showing that a chromosome has not completed replication at the time of anaphase onset. This has made some of the hypothesis difficult to prove.

## The role of the Smc5/6 complex in genome replication

The Smc5–Smc6 complex is, together with cohesin and condensin, one of the three essential eukaryotic SMC complexes. Besides the core Smc5 and Smc6 proteins, [28] the complex has six non-Smc subunits, named Nse1–6 [29,30]. The Smc5–Smc6 complex has been shown to have a role in DNA repair, as well as an essential function. Smc5–Smc6 participates in an undefined step of homologous recombination [31,32]; and it is recruited to DNA double strand breaks to promote sister chromatid recombination [33,34]. Recent work using *smc6 *temperature sensitive alleles has shown that the complex is required for the correct disjunction of the rDNA locus [32]. Mutant cells missegregate regions in chromosome XII distal to rDNA but not proximal. More recently we have shown that the function of the Smc5–Smc6 complex is important for coping with the replication program in rDNA [35]. *smc6–9 *mutant cells enter anaphase on time, as judged by spindle elongation, but nevertheless fail to complete rDNA (and hence chromosome XII) replication. The function of the Smc5–Smc6 complex is specifically required during DNA replication and is no longer required after a metaphase arrest to promote resolution of the rDNA locus. The Smc5–Smc6 function in rDNA replication seems to be two-fold. On one hand it helps forks to traverse through protein-DNA complexes in regions of ongoing transcription; consistent with this, inactivating RNA Pol I transcription or eliminating the presence of RFB suppresses the rDNA missegregation defects in these mutants. On the other hand, it prevents the toxicity of recombination machinery thus avoiding recombination-induced missegregation [36]. During S phase at the restrictive temperature, *smc6–9 *mutant cells display an increase in the number of certain replication intermediates: ongoing forks, forks arrested at the RFB, termination structures and X-shaped DNA. If left unresolved, these structures can prevent sister chromatid resolution and nucleolar segregation during anaphase. While *smc6–9 *cells accumulate 2-times more structures in the rDNA during S phase, the levels of these intermediates is much higher in a metaphase arrest [35], indicating that they are normally removed in wild type cells but persist in *smc6–9 *mutants. *smc6–9 *cells thus suffer a delay in rDNA replication. The accumulation of forks arrested at the RFB is dependent on Fob1 activity, and it most probably reflects the inability of *smc6–9 *mutants to release the Fob1 protein when the replisome is blocked in front of RFB sequences. In this sense, the Smc5–Smc6 complex would have a function similar to the Rrm3 helicase [19]. Rrm3 has been recently shown to be recruited to paused replisomes and to counteract the activity of the Tof1-Csm3 complex [20,37]. However, and in contrast to Smc5–Smc6, Rrm3 is not essential and no nucleolar segregation defects have been reported so far for the *rrm3Δ *mutant, indicating that the Smc5–Smc6 complex has additional roles.

Besides Fob1, two other factors contribute to *smc6–9 *problems in rDNA segregation: high levels of RNA Pol I transcription and *RAD52*-dependent recombination. Genetic analysis of double and triple mutants indicates that the three activities independently contribute to rDNA missegregation in *smc6–9 *mutant cells: none of these processes falls in the same epistatic group for rDNA missegregation. The fact that RNA Pol I transcription, as Fob1 activity, also negatively affects rDNA segregation in *smc6–9 *cells, supports the idea that Smc5–Smc6 function is required to replicate across certain DNA-protein structures. Partial overlapping between these activities cannot be discarded, since the RFB is required to promote inter-repeat recombination [10], RNA Pol I transcription also stimulates recombination [38], and there appears to be a link between transcription and origin firing in the rDNA [39]. How does recombination affect *smc6–9 *cells? One possibility is that replication fork progression defects in *smc6–9 *mutant cells generate structures that are recognized and used by recombination to create more lethal intermediates. However, recombination only slightly contributes to rDNA replication termination defects in *smc5–smc6 *mutants: chromosome XII fails to complete replication before anaphase in a double *smc6–9 rad52Δ *mutant, similar to a single *smc6–9 *mutant. This correlates with the minor suppression of rDNA missegregation by elimination of *RAD52*-mediated recombination and supports the notion that rDNA missegregation is only partially caused by the presence of recombination intermediates at the time of segregation.

## What do Smc5/6 mutants say about the S phase checkpoint?

We have shown that a delay in rDNA replication of *smc5–smc6 *mutants leads to mitosis before cells finish rDNA replication [35]. Importantly, *smc5–smc6 *mutants display proficient checkpoint responses, as cells duly arrest cell cycle progression in response to hydroxyurea, MMS or DSBs (inside and outside of the rDNA array), and do activate Rad53 in all these cases [35]. This indicates that *smc5–smc6 *mutants do not have an S phase checkpoint mutant phenotype and hence do not fit into the "S phase checkpoint gene" category. In *S. pombe*, *smc5–smc6 *mutants also properly arrest the cell cycle and activate checkpoint pathways in response to DNA damage with kinetics identical to those of wild type cells [40,41]. These results, together with ours, suggest that the Smc5–Smc6 complex is not involved in the S phase checkpoint response. At present, we cannot discard the possibility that the Smc5–Smc6 complex is part of a new checkpoint that monitors its own function, so that *smc5–smc6 *mutants are not only defective in replication through difficult templates, but also deficient in signaling such defects to a checkpoint pathway. Since any gene with repair activity could conceptually be described in these terms, this possibility sounds remote. Rather, our results, and those from other authors [42,43], suggest that the Smc5–Smc6 complex is involved in a DNA repair pathway, and its failure is not under checkpoints surveillance.

Our observations further indicate that yeast cells do not monitor ongoing replication and depend on the temporal separation of the two processes to achieve replication completion before anaphase entry. Therefore, it seems that, at least for rDNA replication, cells rely on the ticking cell cycle clock, rather than on checkpoint pathways. It is highly probable some of the other mechanisms described above operate during S phase, such as presence of unlicensed origins or absence of centromere replication (Figure 1).

It is also worth noting that the replication defects we have observed in *smc6–9 *mutants are restricted to the rDNA array. This is due to the presence of multiple obstacles to advancing replication forks in this locus, such as RFBs, and high levels of RNA polymerase transcription. The yeast rDNA is often viewed as a 'special' locus, because of these and other oddities. It is conceivable that some of these peculiarities might include being refractory to replication completion checkpoints. But this does not necessarily implicate that the rest of the genome has mechanisms to monitor fork progression. Proof for the existence of such a mechanism in regions other than the rDNA still awaits. It is worth noting that RFBs have been described in the rDNA array of different organisms, from yeast to plants, frogs, mice and humans [7,8,44-46]. Therefore, even if restricted to the rDNA locus, the absence of a replication completion checkpoint will be of special interest for the maintenance of genome integrity. Since the Smc5–Smc6 complex is essential not only for its rDNA function, it is also possible that it will have a similar role during DNA replication of all the genome. It will be interesting to further explore the role of the Smc5–Smc6 complex in response to blocks in replication fork progression, and its relation to chromosome dynamics and integrity.
